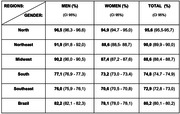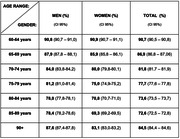# Undetected dementia in Brazil

**DOI:** 10.1002/alz.093303

**Published:** 2025-01-09

**Authors:** Andrew Christopher Miguel, Matheus Ghossain Barbosa, Sonia Brucki, Paulo Caramelli, Jerson Laks, Ricardo Nitrini, Claudia Kimie Suemoto, Cleusa P Ferri

**Affiliations:** ^1^ Universidade Federal de São Paulo, São Paulo, São Paulo Brazil; ^2^ Universidade Federal de São Paulo (UNIFESP), São Paulo, São Paulo/SP Brazil; ^3^ University of São Paulo Medical School, São Paulo Brazil; ^4^ Faculty of Medicine ‐ Universidade Federal de Minas Gerais, Belo Horizonte Brazil; ^5^ Federal University of Rio de Janeiro, Rio de Janeiro Brazil; ^6^ University of São Paulo Medical School, São Paulo, São Paulo Brazil; ^7^ Division of Geriatrics, University of São Paulo Medical School, São Paulo, São Paulo Brazil; ^8^ Federal University of Sao Paulo (UNIFESP), Sao Paulo Brazil

## Abstract

**Background:**

Despite the increasing number of people with dementia, it remains underdetected worldwide, even in high‐income countries. In Brazil, the number of people with dementia is expected to triple by 2050 and diagnosis can be challenging, contributing to high and growing rates of underdiagnosis. At the moment, there is no national estimate of undetected dementia.

**Methods:**

We estimate the proportion of people with dementia (PWD) who are not diagnosed in relation to the total number of PWD estimated by a recent Delphi Consensus. Diagnosed individuals were estimated through the dispensation of anticholinesterases drugs (AChE) in the Unified Health System (SUS), in 2022, for mild and moderate stages of Alzheimer’s Disease (AD), obtained at ftp://ftp.datasus.gov.br, which follows the Ministry of Health national clinical protocol. National literature was consulted for: (i) the proportion of people with mild and moderate AD.; (ii) the proportion of those who obtain AChE from SUS, from the private sector or from both; (iii) the proportion of those who do not take AChE; and (iv) the proportion of AD related to other dementias. We assumed that the underdetection rates of AD would be similar to other dementias and that 70% of the diagnosed individuals with AD obtain AChE from SUS.

**Results:**

More than 80% of the PWD 60+ are undetected (80,2%). Regionally, the North has the highest rates (95,6%), followed by Northeast (90,0%), Midwest (88,6%), South (74,8%) and Southeast (72,9%). Underdiagnosis was higher among men than women (82,2% versus 78,1%) and among the youngest age groups: 60‐64 years (90,7%), decreasing between 65‐69 years (86,9%), 70‐74 years (81,8%) and increasing again in the oldest group 90+ (84,5%).

**Conclusion:**

The underdiagnosis of dementia in Brazil is among the highest in the world. Populational aging trends and higher rates in younger individuals are of concern due to the importance of timely diagnosis. Gender and regional disparities also need to be considered in specific health policies, to increase not only diagnosis rates, but provide adequate support to PWD and their families.